# Neuronal Activity Stimulated by Liquid Substrates Injection at Zusanli (ST36) Acupoint: The Possible Mechanism of *Aquapuncture*

**DOI:** 10.1155/2014/627342

**Published:** 2014-03-06

**Authors:** Chun-Yen Chen, Chao-Nan Lin, Rey-Shyong Chern, Yu-Chuan Tsai, Yung-Hsien Chang, Chi-Hsien Chien

**Affiliations:** ^1^Department of Cell Biology and Anatomy, College of Medicine, National Cheng-Kung University, Tainan 701, Taiwan; ^2^Graduate Institute and Department of Veterinary Medicine, College of Veterinary Medicine, National Pingtung University of Science and Technology, 1 Shuefu Road, Neipu, Pingtung 912, Taiwan; ^3^Department of Anesthesiology, National Cheng Kung University College of Medicine and Hospital, Tainan 701, Taiwan; ^4^Graduate Institute of Chinese Medical Science, China Medical University, Taichung 404, Taiwan

## Abstract

*Aquapuncture* is a modified acupuncture technique and it is generally accepted that it has a greater therapeutic effect than acupuncture because of the combination of the acupoint stimulation and the pharmacological effect of the drugs. However, to date, the mechanisms underlying the effects of *aquapuncture* remain unclear. We hypothesized that both the change in the local spatial configuration and the substrate stimulation of *aquapuncture* would activate neuronal signaling. Thus, bee venom, normal saline, and vitamins B1 and B12 were injected into a Zusanli (ST36) acupoint as substrate of *aquapuncture*, whereas a dry needle was inserted into ST36 as a control. After *aquapuncture*, activated neurons expressing Fos protein were mainly observed in the dorsal horn of the spinal cord in lumbar segments L3–5, with the distribution nearly identical among all groups. However, the bee venom injection induced significantly more Fos-expressing neurons than the other substrates. Based on these data, we suggest that changes in the spatial configuration of the acupoint activate neuronal signaling and that bee venom may further strengthen this neuronal activity. In conclusion, the mechanisms for the effects of *aquapuncture* appear to be the spatial configuration changes occurring within the acupoint and the ability of injected substrates to stimulate neuronal activity.

## 1. Introduction

Acupuncture has been widely used for more than 2000 years in China as an integral part of traditional Chinese medicine in the treatment and prevention of diseases by stimulating specific acupoints [1]. Acupuncture can induce multiple biological responses through the activation of the neuronal system, and the therapeutic benefits of acupuncture treatment have been proven [2–6]. Among several acupuncture techniques, dry needle acupuncture is accomplished by inserting a needle into the acupoint and twisting the needle to achieve a needled sensation. In addition to dry needle stimulation, there are many ways to stimulate an acupoint, including *aquapuncture*, electroacupuncture, acupressure, and even moxibustion.

*Aquapuncture* is an acupoint stimulating technique in which a liquid agent is injected into the acupoint. *Aquapuncture* organically combines acupoint stimulation and the pharmacological effect of drugs with greater therapeutic benefit compared with either routine acupuncture or intramuscular drug injections [7]. Previous studies have revealed that *aquapuncture* can be used for the treatment of early postoperative inflammatory small bowel obstruction, muscle spasticity, chronic gastritis, and even lumbar intervertebral disc herniation [8–11]. The various substrates used for injection include bee venom, normal saline, vitamin B1, vitamin B12, botulinum toxin A, neostigmine, and medicinal herbs [8–10, 12–17]. Although *aquapuncture* has been widely applied clinically, little is known about its therapeutic mechanism of action. During the acupoint *aquapuncture* treatment, the injected liquid substrate causes local spatial configuration changes. Both the spatial configuration changes and the liquid substrate stimulate the acupoint and activate the neuronal system. Our previous studies as well as others suggest that acupuncture regulates physiological functions through activation of the neuronal system [4–6, 18, 19]. Moreover, the activated neuronal system has been proven to play an important role in regulating the physiological functions induced by acupuncture [6, 18, 20]. Therefore, we postulated that both the spatial configuration changes and the substrate stimulation in *aquapuncture* play important roles in the transmission of the acupuncture signal.

To investigate the mechanism of action for *aquapuncture*, we used Fos expression as a neuronal activity marker. Fos protein is an immediate-early gene transcription factor induced by short-term signals that alters target gene expression, causing long-term changes in the cellular phenotype [21]. Fos staining has been used to map activated neural cells after stimulation by various agents [22, 23]. The expression of immediate-early gene transcription factors such as Fos protein has been examined in acupuncture studies [18, 24]. At least one previous study showed that intense or prolonged stimulation increases the number of neurons expressing Fos [25]. Therefore, the number and location of the neurons expressing Fos protein in the spinal dorsal horn were examined to investigate the mechanism of the effect of *aquapuncture* after various liquid agents were injected into Zusanli (ST36). The liquid agents we used were bee venom, normal saline, vitamin B1, and vitamin B12, and dry needle stimulation was used as a control group. The Zusanli (ST36) acupoint, which is widely employed in the treatment of gastrointestinal diseases, has a defined anatomical location and innervation and was chosen for use in this study. Following the changes in spatial configuration and those induced by the substrates of *aquapuncture*, neurons in the dorsal horn of the spinal cord become activated and express Fos protein. The aim of this study was to elucidate the mechanism of actions of *aquapuncture* by evaluating the number and distribution of neurons expressing Fos protein.

## 2. Materials and Methods

The study protocol was approved by our institution's Animal Care and Use Committee, and all experiments were conducted in accordance with the guidelines of animal care from the National Institutes of Health and the International Association.

### 2.1. Animals

Adult male Sprague-Dawley rats (250–350 g) were used. The animals were housed in an environment with a 12 h light-dark cycle and free access to standard food and tap water.

### 2.2. *Aquapuncture* with Various Substrates

The rats were divided into six groups and anesthetized with ketamine (1 mL/kg) injected intraperitoneally. For each substrate-treated group, the substrates—0.1 mL of 1% bee venom (*n* = 4), normal saline (*n* = 3), vitamin B1 (*n* = 7), and vitamin B12 (*n* = 4)—were injected intramuscularly into the left Zusanli (ST36), which is located on the lateral side of the stifle joint adjacent to the anterior tubercle of the tibia. A dry needle (with no substrate injected) was used in one control group; no needles were inserted into ST36 in the sham control group. The anatomical location of the ST36 acupoint is equivalent in rats and humans. For each substrate-treated group, the substrates—0.1 mL of 1% bee venom (*n* = 4), normal saline (*n* = 3), vitamin B1 (*n* = 7), and vitamin B12 (*n* = 4)—were injected intramuscularly into the left Zusanli (ST36), which is located on the lateral side of the stifle joint adjacent to the anterior tubercle of the tibia. Needles were inserted without rotation into the left ST36 of rats in the dry needle group (*n* = 3). The rats were sacrificed 2 hours after substrate injection or dry needle insertion. For sacrifice, the animals were anesthetized and perfused intracardially with 250 mL of saline followed by 1000 mL of 4% paraformaldehyde in 0.1 M phosphate buffered solution (PBS). Spinal cord segments L3–5 were removed.

### 2.3. Fos Immunohistochemistry and Analysis

Spinal cords were postfixed up to 4 hours in 4% paraformaldehyde and then cryoprotected in a 10, 20, and 30% sucrose solution. Serial 30 *μ*m thick transverse sections from all spinal cords were cut with a cryomicrotome. All sections containing ganglia and every five sections from other regions were collected in 0.01 M PBS. The floating sections were washed for 30 min (10 min, 3 times) and incubated with blocking solution (5% normal goat serum, 0.05% Triton X-100, 3% BSA in 0.1 M PB) for 1 hour. The sections were washed and incubated with the primary antibody (anti-FOS rabbit IgG, 1 : 2000, SANTA CRUZ) in blocking solution for 72 hours at 4°C. After incubation, the sections were rinsed and incubated for 1 hour at 25°C with secondary antibody (biotin-conjugated goat anti-rabbit IgG, 1 : 500, Jackson) in blocking solution. The sections were washed three times for 30 min and then processed with an ABC kit (Vector) using a 1 hour incubation. After rinsing, the sections were developed using the glucose-oxidase-nickel-DAB (GOD) method, mounted on gelatin-coated slides and cover slipped with mounting medium. Fos immunoreactive neurons were counted using bright-field microscopy.

## 3. Results

**Figure 1:**
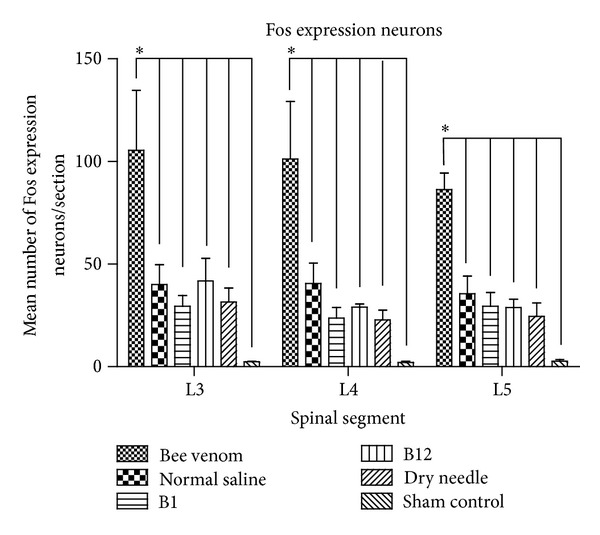
The mean number of Fos expression neurons in L3–5 spinal dorsal horn between bee venom, normal saline, vitamin B1, vitamin B12 injection, and dry needle groups. Fos expression neurons of bee venom group were significantly more than normal saline, vitamins B1 and B12, dry needle, and sham control groups. There is no markedly difference between normal saline, vitamins B1 and B12, and dry needle groups. The sham control group expressed significantly lesser Fos expression neurons than the other groups. *P < 0.05.

**Figure 2:**
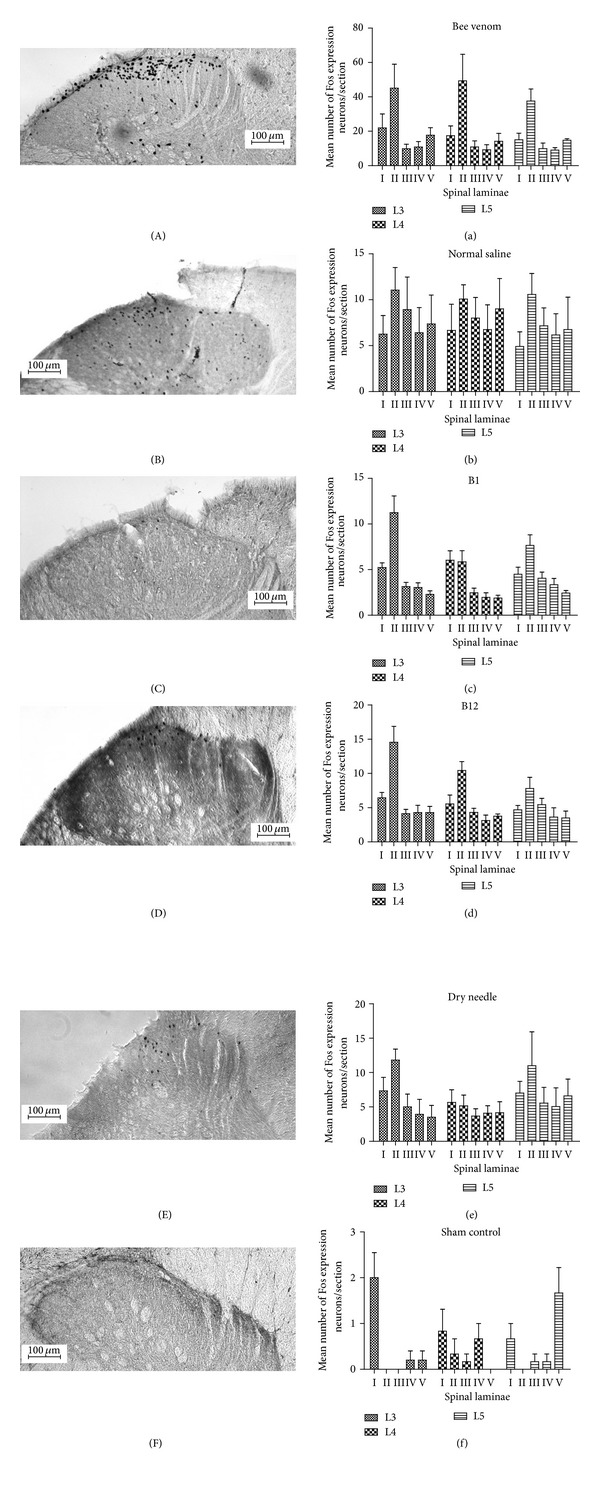
Distribution of Fos expression in laminae I~V of 3rd, 4th, and 5th lumbar spinal cord after the ST36 acupoint stimulation. (A, a) Bee venom group; (B, b) normal saline group; (C, c) vitamin B1 group; (D, d) vitamin B12 group; (E, e) dry needle insertion group; (F, f) sham control group; (scale bar: 100 um).

Several different liquid substrates, including 1% bee venom, normal saline, and vitamins B1 and B12, were injected into the ST36 acupoint. A dry needle insertion was used in one control group, and a sham group was used as the negative control. Two hours later, the expression of Fos in the ipsilateral spinal dorsal horn neurons of the 3rd, 4th, and 5th lumbar segments was investigated immunohistochemically. Fos-expressing neurons were observed in rats exposed to bee venom (L3: 105.4 ± 29.4, L4: 101.1 ± 28.1, L5: 86.3 ± 8.0, sum: 292.8 ± 60.7), normal saline (L3: 40.0 ± 9.7, L4: 40.5 ± 10.0, L5: 35.6 ± 8.6, sum: 116.1 ± 28.2), vitamin B1 (L3: 29.4 ± 5.3, L4: 23.7 ± 5.2, L5: 29.4 ± 6.8, sum: 82.4 ± 16.8), vitamin B12 (L3: 41.8 ± 11.0, L4: 29.0 ± 1.6, L5: 28.8 ± 4.2, sum: 99.5 ± 15.5), and dry needle (L3: 31.6 ± 6.7, L4: 22.8 ± 4.9, L5: 24.5 ± 6.5, sum: 78.8 ± 3.8) (Table 1, Figure 1). By contrast, only a few neurons expressing FOS immunoreactivity were detected in the sham control group (L3: 2.4 ± 0.5, L4: 2.0 ± 1.7, L5: 2.7 ± 2.1, sum: 7.1 ± 0.3) (Table 1, Figures 1, 2(F), and 2(f)). The neurons from animals in the five groups expressing Fos immunoreactivity were distributed primarily in the intermedial zone of the dorsal horn of the spinal cord (Figures 2(A), 2(B), 2(C), 2(D), and 2(E)). Fos-expressing neurons were observed in laminae I−V of the dorsal horn, but mainly in lamina II (Figures 2(a), 2(b), 2(c), 2(d), and 2(e)), with a similar distribution pattern across all groups (Figure 2).

The various liquid substrates injected and the dry needle insertion into Zusanli (ST36) induced Fos expression in the L3, L4, and L5 segments of the spinal cord. The group injected with bee venom had more Fos-positive neurons in the dorsal horn than the other groups (Table 1, Figure 1). All treated groups showed more Fos-expressing neurons than the sham control group. In addition, the Fos-positive neurons in all groups were primarily distributed in lamina II of spinal dorsal horn (Figure 2).

## 4. Discussion

Fos expression has been used to detect neuronal activation in many different studies, including those examining the acupuncture pathway [6, 18, 24, 26–32]. Previous studies using acupuncture or electroacupuncture have shown that the Zusanli (ST36) acupoint is innervated by spinal cord segments L3–5 [33]. The neuronal innervation of the Zusanli (ST36) acupoint has been thoroughly characterized and may inform our result that the spinal dorsal horn neurons in lumbar segments L3, L4, and L5 were activated by *aquapuncture* injection into the Zusanli (ST36) acupoint. The study by Kim et al. in 2012 showed an increased expression of axonal growth-associated protein (GAP-43) and phospho-Erk1/2 in the DRG neurons of L4 and L5 after electroacupuncture stimulation of ST36. That study suggested that acupuncture stimulation generates neuronal responses in the autonomic nervous system via the activation of the somatosensory pathway [18]. In our study, most of the Fos-positive neurons were located in the 3rd, 4th and 5th lumbar segments following the injection of various substrates or a dry needle inserted into the ST36 acupoint. Based on these results, we suggest that *aquapuncture* stimulation may not only activate the somatosensory pathway but also induce physiological effects similar to those of other acupuncture methods.

The effects of *aquapuncture* included the acupoint stimulation as well as the pharmacological actions of the injected substrates. Normal saline has been injected into acupoints to treat chronic back pain in horses [17]. Injections of vitamin B12 into acupoints have been used as a simple and effective method for the treatment of facial verruca plana [14]. Acupoint injections with vitamin B1 also have proven effective for the treatment of urticaria [15]. In addition, injections of bee venom into Zusanli (ST36) can modulate methamphetamine-induced hyperactivity and hyperthermia [31]. The liquid agents in those studies were not specific for those maladies, suggesting that the therapeutic effect of *aquapuncture* may be mediated mainly through changes in the spatial configuration of the acupoint. The present study showed that the distribution of Fos-expressing neurons following the dry needle insertion was similar to that following the injection of liquid and was observed primarily in lamina II of the dorsal horn of the spinal cord (Figure 2). These results suggest that bee venom, normal saline, and vitamins B1 and B12 injections stimulate the acupoint and activate the same acupuncture pathway regardless of their distinct pharmacological effects.

The acupoint stimulation of *aquapuncture* includes stimulation induced by the liquid substrate itself as well as by changes in the spatial configuration of the acupoint caused by a liquid injection. In our study, the volume of each liquid injected was the same and induced similar spatial configuration changes. The injections of normal saline, vitamin B1, or vitamin B12 induced no significant differences in the number of Fos-expressing neurons. By contrast, the bee venom injection induced a greater number of Fos-expressing neurons than the other agents (Figure 1). The main ingredients in bee venom, histamine and melittin, can activate neural fibers [34] and spinal dorsal horn neurons. Thus, given equal injection volumes, we suggest that bee venom may have a greater effect than normal saline, vitamin B1, or vitamin B12 in stimulating the acupoint and activating the neuronal system.

It is generally accepted that the effects of *aquapuncture* are attributable to the integration of the acupoint stimulation and the pharmacological actions of substrates [7]. Our study showed that the different substrates for *aquapuncture* induced neuronal activation at various intensities, but that the distribution of these activated neurons was virtually the same as that for acupuncture. Our study suggests that spatial configuration changes in the acupointcombined with the neuronal activity enhancement induced by the injected substrates likely mediate the effects of *aquapuncture*.

## Figures and Tables

**Table 1:** Substrates of *aquapuncture* injection and dry needle insertion into ST36 induce Fos expression in 3rd, 4th, and 5th lumbar spinal dorsal horn.

	Bee venom	Normal saline	Vitamin B1	Vitamin B12	Dry needle	Sham control
L3	105.4 ± 29.4	40.0 ± 9.7	29.4 ± 5.3	41.8 ± 11.0	31.6 ± 6.7	2.4 ± 0.5
L4	101.1 ± 28.1	40.5 ± 10.0	23.7 ± 5.2	29.0 ± 1.6	22.8 ± 4.9	2.0 ± 1.7
L5	86.3 ± 8.0	35.6 ± 8.6	29.4 ± 6.8	28.8 ± 4.2	24.5 ± 6.5	2.7 ± 2.1

Sum	292.8 ± 60.7	116.1 ± 28.2	82.4 ± 16.8	99.5 ± 15.5	78.8 ± 3.8	7.1 ± 0.3

## References

[1] World Health Organization (1993). *Regional Office for the Western Pacific. Standard Acupuncture Nomenclature: A Brief Explanation of 361 Classical Acupuncture
Point Names and Their Multilingual Comparative List*.

[2] Chao DM, Shen LL, Tjen-A-Looi S, Pitsillides KF, Li P, Longhurst JC (1999). Naloxone reverses inhibitory effect of electroacupuncture on sympathetic cardiovascular reflex responses. *American Journal of Physiology*.

[3] Moazzami A, Tjen-A-Looi SC, Guo Z-L, Longhurst JC (2010). Serotonergic projection from nucleus raphe pallidus to rostral ventrolateral medulla modulates cardiovascular reflex responses during acupuncture. *Journal of Applied Physiology*.

[4] Peng L (2002). Neural mechanisms of the effect of acupuncture on cardiovascular diseases. *International Congress Series*.

[5] Chien C-H, Shieh J-Y, Liao M-H, Ling E-A, Wen C-Y (1998). Neuronal connections between the auricular skin and the sympathetic pre- and postganglionic neurons of the dog as studied by using pseudorabies virus. *Neuroscience Research*.

[6] Chen C-Y, Chern R-S, Liao M-H, Chang Y-H, Hsu J-YC, Chien C-H (2013). The possible neuronal mechanism of acupuncture: morphological evidence of the neuronal connection between groin A-shi point and uterus. *Evidence-Based Complementary and Alternative Medicine*.

[7] Zhang Y, Chen F, Wu S (2007). Clinical observation on O3 acupoint injection for treatment of low back pain. *Zhongguo zhen jiu = Chinese acupuncture & moxibustion*.

[8] Shen L-P, Guan J, Ding K-Y (2010). Clinical observation on electroacupuncture combined with acupoint injection for treatment of early postoperative inflammatory intestinal obstruction. *Zhongguo Zhen Jiu*.

[9] Xing S-T, Wang D, Wen X-H (2010). Clinical research of electroacupuncture combined with acupoint-injection of botulinum toxin A in treating the muscle spasticity by spinal cord injury. *Zhongguo Gu Shang*.

[10] Zhang Y-B, Yan C-Y (2010). Clinical observation on acupoint injection therapy for chronic gastritis of gastric blood stasis type. *Zhongguo Zhen Jiu*.

[11] Zou R, Xu Y, Zhang H-X (2009). Evaluation on analgesic effect of electroacupuncture combined with acupoint-injection in treating lumbar intervertebral disc herniation. *Zhongguo Gu Shang*.

[12] Yoon S-Y, Roh D-H, Kwon Y-B (2009). Acupoint stimulation with diluted bee venom (apipuncture) potentiates the analgesic effect of intrathecal clonidine in the rodent formalin test and in a neuropathic pain model. *Journal of Pain*.

[13] Wang Q, Xiong J-X, Pan W-Y (2006). Observation on treatment of chronic pelvic inflammatory with point injection combined with ultra-laser radiation. *Zhongguo Zhen Jiu*.

[14] Hu Y, Zhang W, Chen L (1998). The effect of Vit. B12 injection into acupoints in the treatment of verruca plana. *Zhonghua Kou Qiang Yi Xue Za Zhi*.

[15] Chen C-J, Yu H-S (1998). Acupuncture treatment of urticaria. *Archives of Dermatology*.

[16] Hameroff SR, Crago B, Blitt D (1981). Comparison of bupivacaine, etidocaine, and saline for trigger-point therapy. *Anesthesia and Analgesia*.

[17] Martin BB, Klide AM (1987). Use of acupuncture for the treatment of chronic back pain in horses: stimulation of acupuncture points with saline solution injections. *Journal of the American Veterinary Medical Association*.

[18] Kim MH, Park YC, Namgung U (2012). Acupuncture-stimulated activation of sensory neurons. *Journal of Acupuncture and Meridian Studies*.

[19] Li P, Pitsillides KF, Rendig SV, Pan H-L, Longhurst JC (1998). Reversal of reflex-induced myocardial ischemia by median nerve stimulation: a feline model of electroacupuncture. *Circulation*.

[20] Gao XY, Li YH, Liu K (2011). Acupuncture-like stimulation at auricular point Heart evokes cardiovascular inhibition via activating the cardiac-related neurons in the nucleus tractus solitarius. *Brain Research*.

[21] Curran T, Morgan JI (1995). Fos: an immediate-early transcription factor in neurons. *Journal of Neurobiology*.

[22] Luo C, Chen J, Li H-L, Li J-S (1998). Spatial and temporal expression of c-Fos protein in the spinal cord of anesthetized rat induced by subcutaneous bee venom injection. *Brain Research*.

[23] Ghanima A, Bennis M, Rampin O (2002). c-Fos expression as endogenous marker of lumbosacral spinal neuron activity in response to vaginocervical-stimulation. *Brain Research Protocols*.

[24] De Medeiros MA, Canteras NS, Suchecki D, Mello LEAM (2003). Analgesia and c-Fos expression in the periaqueductal gray induced by electroacupuncture at the Zusanli point in rats. *Brain Research*.

[25] Yi DK, Barr GA (1995). The induction of Fos-like immunoreactivity by noxious thermal, mechanical and chemical stimuli in the lumbar spinal cord of infant rats. *Pain*.

[26] Martínez V, Wang L, Taché Y (2006). Proximal colon distension induces Fos expression in the brain and inhibits gastric emptying through capsaicin-sensitive pathways in conscious rats. *Brain Research*.

[27] Mönnikes H, Rüter J, König M (2003). Differential induction of c-fos expression in brain nuclei by noxious and non-noxious colonic distension: role of afferent C-fibers and 5-HT3 receptors. *Brain Research*.

[28] Pinto M, Lima D, Tavares I (2006). Correlation of noxious evoked c-fos expression in areas of the somatosensory system during chronic pain: involvement of spino-medullary and intra-medullary connections. *Neuroscience Letters*.

[29] Roh D-H, Kim H-W, Yoon S-Y (2006). Bee venom injection significantly reduces nociceptive behavior in the mouse formalin test via capsaicin-insensitive afferents. *Journal of Pain*.

[30] Tavares I, Lima D, Coimbra A (1993). Neurons in the superficial dorsal horn of the rat spinal cord projecting to the medullary ventrolateral reticular formation express c-fos after noxious stimulation of the skin. *Brain Research*.

[31] Kim KW, Kim HW, Li J, Kwon YB (2011). Effect of bee venom acupuncture on methamphetamine-induced hyperactivity, hyperthermia and Fos expression in mice. *Brain Research Bulletin*.

[32] Qi DB, Li WM (2012). Effects of electroacupuncture on expression of c-fos protein in the spinal dorsal horn of rats with chronic visceral hyperalgesia. *Zhong Xi Yi Jie He Xue Bao*.

[33] Kim H-N, Kim Y-R, Jang J-Y, Shin H-K, Choi B-T (2012). Effects of electroacupuncture on N-methyl-D-aspartate receptor-related signaling pathway in the spinal cord of normal rats. *Evidence-Based Complementary and Alternative Medicine*.

[34] Habermann E, Bücherl W, Buckley EE (1971). Chemistry, pharmacology, and toxicology of bee, wasp, and hornet venoms. *Venomous Animals and Their Toxins: Volume III, Venomous Invertebrates*.

